# Supplementary material for: Discovery of 17 conserved structural RNAs in fungi
Source: Nucleic Acids Res. 2021 Jun 4;49(11):6128–43. doi: 10.1093/nar/gkab355 (PMC8216456; doi:10.1093/nar/gkab355)
Supplement: gkab355_Supplemental_Files [file gkab355_supplemental_files.zip › supplemental_data.tar.gz!/supplemental_data/supplement/supplement.pdf]

Discovery of 17 conserved structural RNAs in fungi

Supplemental Materials

William Gao, Thomas A. Jones, and Elena Rivas

March 23, 2021

Supplemental Tables

Intergenic regions (unflanked mode)

Species	Nuclear genome size (Mb)	# IGRs	# windows under 100 nt	# windows w/o final nhmmer	# windows w/ final nhmmer
S. cerevisiae	12.1	6699	589	271	7031
C. albicans	14.3	6263	639	3069	7180
N. crassa	40.5	9173	555	10714	22664
A. fumigatus	29.4	11709	600	2977	22484
S. pombe	12.6	4401	975	178	4320

Untranslated regions / introns (flanked mode)

Species	# protein-coding genes	# introns under 100 nt	# introns w/o final nhmmer	# introns w/ final nhmmer	# UTRs under 100 nt	# UTRs w/o final nhmmer	# UTRs w/ final nhmmer
S. cerevisiae	6572	110	9	190	870	39	10748
C. albicans	6245	237	0	165	1227	356	10612
N. crassa	10381	13908	55	6044	1069	517	16445
A. fumigatus	9859	16208	8	2413	723	797	17496
S. pombe	7097	634	0	236	1332	158	6569

Table S1. **Details of fungal screen.** For each of the five query genomes, we report the number of intergenic regions subject to the unflanked mode, and the number of UTRs and intronic sequences subject to the flanking mode. Query sequences shorter than 100 nt were removed from the analysis, because an exact match of 100 nt was empirically determined for our 1371 fungal genome database to have an E-value close to the E-value threshold of 10^{-10} for the first iteration. For the remaining sequences, we report the number with alignments obtained after the 3-fold iterative homology search performed using nhmmer. Almost all of the sequences that do not have a final nhmmer alignment are from low complexity or highly repetitive regions that were not provided in the genome annotations. UTRs and intergenic regions were only defined for regions that are not overlapping with any other annotations. *S. pombe* is an genome where a significant portion of annotations are overlapping, resulting in fewer UTRs and intergenic regions than expected for the number of annotated protein-coding genes. Refer to Fig. S2 for an example of a genomic region and how annotations were used to identify IGRs, introns, and UTRs.

Structural RNA	Model Organism(s)	Coordinates	Size (nts)	Closest gene
01 H/ACA snoRNA 1	*C. albicans*	chr2:424209-424497	289	SAM37: 422849-423919
02 H/ACA snoRNA 2	*C. albicans*	chrR:2099614-2100061	448	FGR46: 2100560-2100198
03 H/ACA snoRNA 3	*A. fumigatus*	chr7:1017260-1017427	168	AFUA_7G04460: 1015962-1017200
04 H/ACA snoRNA intronic	*A. fumigatus*	chr2:3571437-3571242	196	AFUA_2G13750: 3571860-3569481
05 conserved intergenic structure 1	*A. fumigatus*<br>*N. crassa*	chr3:2027363-2027443<br>chr1:8347842-8347751	81<br>92	AFUA_3G07970: 2028224-2026400<br>NCU03041: 8346947-8349780
06 conserved intergenic structure 2	*A. fumigatus*	chr1:4003534-4003702	169	AFUA_1G14940: 4004644-4007079
07 conserved intergenic structure 3	*A. fumigatus*<br>*N. crassa*	chr2:899227-899360<br>chr2:2737780-2737909	134<br>130	AFUA_2G03420: 899213-901323<br>NCU00133: 2737574-2740109
08 conserved intergenic structure 4	*A. fumigatus*<br>*N. crassa*	chr5:3359121-3359194<br>chr5:2294613-2294542	74<br>72	AFUA_5G12830: 3360387-3359565<br>NCU03749: 2292401-2294704
09 *RPL12* $5'$ UTR	*A. fumigatus*	chr1:988401-988346	56	AFUA_1G03390: 988333-987678
10 *RPS13* intron	C. albicans	chr6:115411-115298	114	RPS13: 105929-103938
11 lysyl-tRNA synthetase intron / $5'$ UTR	A. fumigatus	chr1:1270323-1270266	58	AFUA_1G04460: 1270442-1268328
12 metacaspase 1 intron	*N. crassa*	chr7:2633707-2633620	88	NCU02400: 2635820-2631309
13 thioredoxin $3'$ UTR	*A. fumigatus*<br>*N. crassa*	chr4:2371534-2371435<br>chr1:2599913-2600026	100<br>114	AFUA_4G09080: 2360133-2358123<br>NCU02520: 2598003-2599740
14 RING finger domain protein $3'$ UTR	*A. fumigatus*<br>*N. crassa*	chr7:1436607-1436525<br>chr2:2021057-2021142	83<br>86	AFUA_7G05850: 1435276-1433710<br>NCU06763: 2019543-2021559
15 thioesterase family protein $3'$ UTR	*A. fumigatus*	chr2:1904008-1904176	169	AFUA_2G07440: 1903479-1903916
16 *GLY1* $3'$ UTR	*S. cerevisiae*	chr5:67409-67247	163	GLY1: 68792-67629
17 *MET13* $3'$ UTR	*C. albicans*	chr3:627997-627954	44	MET13: 629936-628092

Table S2. **Details of the 17 fungal structural RNAs.** The NCBI accessions for the first chromosome of the five query alignments are the following: *S. cerevisiae* (BK006935.2), *C. albicans* (CP017623.1), *N. crassa* (NC_026501.1), *A. fumigatus* (NC_007194.1), and *S. pombe* (CU329670.1).

Table S3. **106 hits removed across the five query genomes.**

genome	Intergenic region chr:start-end	Alignment avg seq len	Rfam hit	Rfam description
A. fumigatus	1:913875-917095	217	RF00001	5S rRNA
A. fumigatus	1:966944-968820	223	RF00001	5S rRNA
A. fumigatus	1:986548-987545	233	RF01269	Small nucleolar RNA snR80
A. fumigatus	1:1387321-1388627	698	RF00009	Nuclear RNase P
A. fumigatus	1:1991130-1991861	330	RF00003	U1 spliceosomal RNA
A. fumigatus	2:4172233-4172658	126	RF00001	5S rRNA
A. fumigatus	2:4534354-4534839	241	RF01261	Small nucleolar RNA snR82
A. fumigatus	3:638227-639225	203	RF00001	5S rRNA
A. fumigatus	3:687949-688947	200	RF00001	5S rRNA
A. fumigatus	3:1089131-1090129	231	RF00001	5S rRNA
A. fumigatus	3:3323928-3325304	183	RF00005	tRNA
A. fumigatus	3:3785055-3785278	196	RF01503	A. fumigatus sRNA Afu 203
A. fumigatus	4:755217-756213	186	RF00001	5S rRNA
A. fumigatus	4:757715-758711	240	RF00001	5S rRNA
A. fumigatus	4:1996559-1997556	282	RF01496	A. fumigatus sRNA Afu 182
A. fumigatus	4:3162957-3163743	188	RF00001	5S rRNA
A. fumigatus	4:3279339-3280517	341	RF00003	U1 spliceosomal RNA
A. fumigatus	5:309691-310445	131	RF00005	tRNA
A. fumigatus	5:2496656-2497518	539	RF01434	Small nucleolar RNA snR3
A. fumigatus	5:2631624-2636010	182	RF02543	Eukaryotic LSU rRNA
A. fumigatus	6:31672-32555	160	RF02543	Eukaryotic LSU rRNA
A. fumigatus	6:1654992-1656125	430	RF00005	tRNA
A. fumigatus	6:1656782-1660223	495	RF00005	tRNA
A. fumigatus	6:2787410-2788874	462	RF01239	Small nucleolar RNA snR49
A. fumigatus	6:3000535-3001451	244	RF00001	5S rRNA
A. fumigatus	6:3477308-3478208	183	RF00005	tRNA
A. fumigatus	7:319031-319850	251	RF01240	Small nucleolar RNA snR85
A. fumigatus	7:662854-664651	342	RF01445	Small nucleolar RNA snR94
A. fumigatus	7:817049-817258	118	RF00005	tRNA
A. fumigatus	8:422221-423216	233	RF00001	5S rRNA
A. fumigatus	8:1542740-1545898	133	RF00005	tRNA
A. fumigatus	8:1792689-1793274	113	RF00001	5S rRNA

genome	Intergenic region chr:start-end	Alignment avg seq len	Rfam hit	Rfam description
C. albicans	1:1095960-1097142	167	RF01245	Small nucleolar RNA snR9
C. albicans	1:1862322-1862510	120	RF00005	tRNA
C. albicans	1:2384577-2386237	219	RF00005	tRNA
C. albicans	1:2587936-2588554	143	RF00005	tRNA
C. albicans	1:2960366-2960890	204	RF01258	Small nucleolar RNA snR10
C. albicans	1:3120122-3120929	359	RF01267	Small nucleolar RNA snR37
C. albicans	2:638517-639334	136	RF00005	tRNA
C. albicans	2:833442-833939	232	RF01260	Small nucleolar RNA snR11
C. albicans	2:1077440-1077328	127	RF00005	tRNA
C. albicans	2:1497591-1498446	244	RF01266	Small nucleolar RNA snR45
C. albicans	2:1620125-1620260	115	RF00005	tRNA
C. albicans	2:2179215-2179654	263	RF01256	Small nucleolar RNA snR43
C. albicans	3:458225-459259	351	RF01269	Small nucleolar RNA snR80
C. albicans	3:681093-681277	155	RF00005	tRNA
C. albicans	3:1734285-1734632	264	RF01243	Small nucleolar RNA snR33
C. albicans	4:902632-903051	118	RF00005	tRNA
C. albicans	4:1251386-1251607	172	RF00005	tRNA
C. albicans	4:1324975-1325487	123	RF00005	tRNA
C. albicans	4:1381379-1382068	213	RF01242	Small nucleolar RNA snR36
C. albicans	5:201169-201945	157	RF00005	tRNA
C. albicans	5:305503-306587	170	RF01960	Eukaryotic SSU rRNA
C. albicans	5:430140-430754	133	RF00005	tRNA
C. albicans	6:171333-172240	267	RF00004	U2 spliceosomal RNA
C. albicans	7:839560-840835	171	RF00005	tRNA
C. albicans	R:807594-808432	152	RF01253	Small nucleolar RNA snR46
C. albicans	R:1164444-1164566	112	RF01255	Small nucleolar RNA snR35
C. albicans	R:1830002-1831039	230	RF01254	Small nucleolar RNA snR34
C. albicans	R:1889053-1891234	557	RF01960	Eukaryotic SSU rRNA
C. albicans	R:1909314-1910064	111	RF00005	tRNA

genome	Intergenic region chr:start-end	Alignment avg seq len	Rfam hit	Rfam description
N. crassa	1:6442-9168	325	RF00005	tRNA
N. crassa	1:238577-239090	172	RF01247	Small nucleolar RNA snR32
N. crassa	1:1363798-1364791	122	RF00001	5S rRNA
N. crassa	1:2887936-2888376	192	RF00009	Nuclear RNase P
N. crassa	1:2907344-2907539	178	RF01513	A. fumigatus snoRNA Afu 335
N. crassa	1:2977600-2980995	143	RF00001	5S rRNA
N. crassa	1:2993018-2994253	129	RF00093	Small nucleolar RNA SNORD18
N. crassa	1:5844823-5845352	101	RF00001	5S rRNA
N. crassa	2:2206339-2206812	122	RF00001	5S rRNA
N. crassa	2:2948495-2949193	482	RF02462	Ascomycota telomerase RNA
N. crassa	2:3211687-3214126	233	RF01512	A. fumigatus sRNA Afu 309
N. crassa	2:3540815-3541813	197	RF00001	5S rRNA
N. crassa	3:3354646-3355643	218	RF01444	Small nucleolar RNA snR92
N. crassa	3:3728570-3729379	146	RF00001	5S rRNA
N. crassa	3:3981904-3982863	170	RF00005	tRNA
N. crassa	3:3983902-3985130	278	RF01242	Small nucleolar RNA snR36
N. crassa	3:4357563-4358022	224	RF01261	Small nucleolar RNA snR82
N. crassa	4:555599-558380	358	RF00005	tRNA
N. crassa	4:3609675-3610402	177	RF00001	5S rRNA
N. crassa	4:3928462-3929454	195	RF00001	5S rRNA
N. crassa	5:90084-91079	162	RF02543	Eukaryotic LSU rRNA
N. crassa	5:416602-419043	220	RF00005	tRNA
N. crassa	5:778325-780046	119	RF00005	tRNA
N. crassa	5:837020-838658	287	RF01434	Small nucleolar RNA snR3
N. crassa	5:2422699-2422964	122	RF00001	5S rRNA
N. crassa	5:5917842-5918648	316	RF00003	U1 spliceosomal RNA
N. crassa	6:323571-324398	310	RF00003	U1 spliceosomal RNA
N. crassa	6:1113628-1114624	276	RF00003	U1 spliceosomal RNA
N. crassa	7:987119-988115	308	RF00003	U1 spliceosomal RNA
N. crassa	7:3654557-3655034	225	RF01248	Small nucleolar RNA snR8
N. crassa	7:4139782-4140779	194	RF00001	5S rRNA

genome	Intergenic region chr:start-end	Alignment avg seq len	Rfam hit	Rfam description
S. cerevisiae	4:158735-159601	196	RF02543	Eukaryotic LSU rRNA
S. cerevisiae	7:2079-2790	147	RF00005	tRNA
S. cerevisiae	12:451963-452438	401	RF02543	Eukaryotic LSU rRNA
S. cerevisiae	12:453131-454122	802	RF02543	Eukaryotic LSU rRNA
S. cerevisiae	12:454534-455180	567	RF02543	Eukaryotic LSU rRNA

genome	Intergenic region chr:start-end	Alignment avg seq len	Rfam hit	Rfam description
S. pombe	1:952610-952873	143	RF00005	tRNA
S. pombe	1:1746469-1746913	127	RF00005	tRNA
S. pombe	1:1845709-1846109	178	RF00001	5S rRNA
S. pombe	1:3157303-3157492	108	RF00005	tRNA
S. pombe	2:323950-324167	124	RF00005	tRNA
S. pombe	2:1602266-1602771	162	RF00005	tRNA
S. pombe	2:2322721-2323014	177	RF00001	5S rRNA
S. pombe	2:2948203-2948644	167	RF00001	5S rRNA
S. pombe	3:814711-815084	284	RF02543	Eukaryotic LSU rRNA

Table S3. Structures that satisfied the selection criteria but had a significant hit to any Rfam families were removed. The list of 106 removed hits includes: 35 rRNAs, 32 tRNAs, 25 snoRNAs, and 15 other RNA genes.

Supplemental Figures

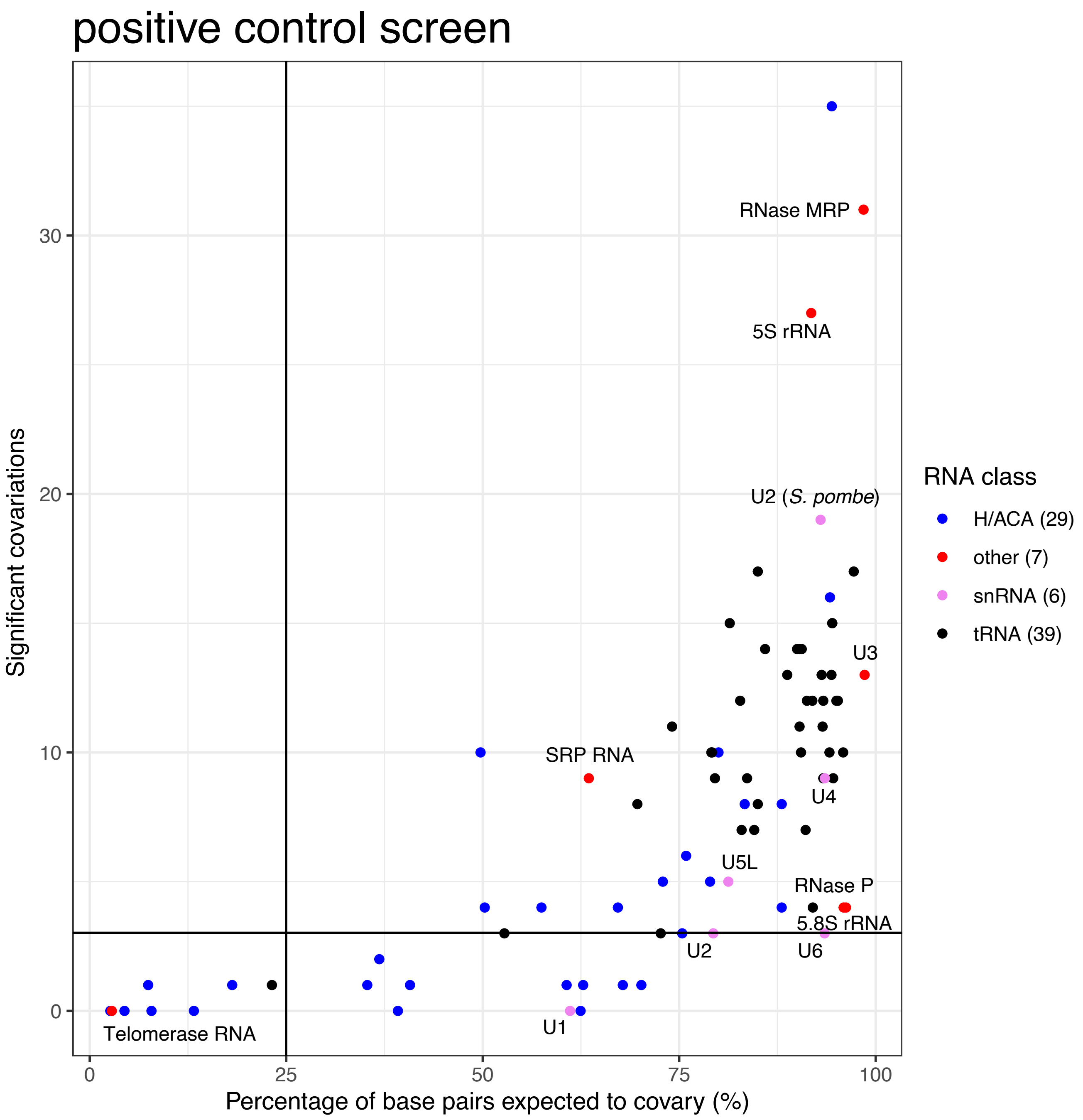

Figure S1: **Results of optimized method in positive controls**. When optimizing our method for discovering structural non-coding RNAs, we tested how two parameters, the number of iterative homology searches and E-value threshold, affected the number of statistically significant covariations (inferred as conserved base pairs) detected in alignments of these known functional ncRNAs. This set of positive controls included 80 structural ncRNA genes from the *S. cerevisiae* genome, including all 29 H/ACA box small nucleolar RNAs (snoRNA), 5S and 5.8S ribosomal RNAs (rRNA), 39 transfer RNAs (tRNA) with one per anticodon, 5 spliceosomal RNAs (snRNA), and 5 other RNAs (RNase MRP, RNase P, SRP RNA, Telomerase RNA, and U3). These sequences were flanked with upstream and downstream intergenic sequences, to simulate a screen for standalone ncRNA genes using the unflanked mode. With the optimized unflanked mode (see Main Fig. 2), 62 out of 80 (78%) ncRNAs have 3 or more conserved base pairs, and only 10 (13%) have fewer than 3 conserved base pairs when more than 25% of base pairs are expected to covary (measure of covariation power). 8 (10%) of these ncRNAs have less than a 25% of base pairs expected to covary, and in all cases, these ncRNAs have low covariation power because homology is only detected in the *Saccharomyces sensu stricto*. In some cases, fewer conserved base pairs are found than expected because nhmmer does not produce alignments that account for secondary structure or because of peculiarities in the query sequence. For example, the U2 sequence from *S. cerevisiae* is unusually large compared to homologs from other fungi (1). Using a sequence more representative of fungal U2s such as the one from *S. pombe* resulted in detection of many more conserved base pairs. All ncRNAs except H/ACA box snoRNAs and tRNAs are labeled on the plot.

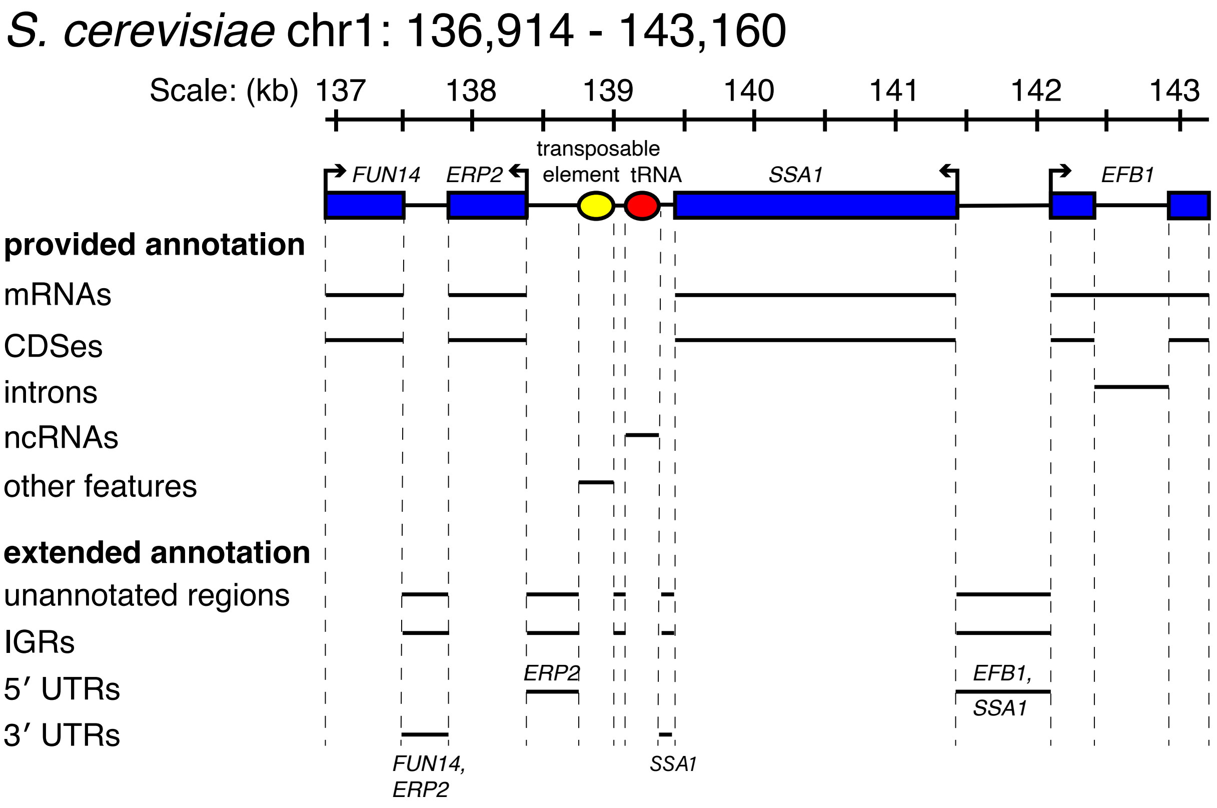

Figure S2: **Example of a genomic region screened**. Here, a 6 kb region of the *S. cerevisiae* genome is shown with annotations from the most recent (01/13/2015) annotation from the *Saccharomyces* Genome Database. The annotations give the coordinates of protein-coding genes, non-coding genes, and other features like transposable elements. The coordinates of introns are also provided. Based on this annotation, we defined intergenic regions (IGRs) as any unannotated regions. If intronic coordinates are explicitly provided (as for *S. cerevisiae* and *S. pombe*), we use those coordinates. In the annotations of the other three query genomes, the intron coordinates are not explicitly defined, so we define them as the intervening sequence between two annotated exons of the same protein-coding gene. If the coordinates of untranslated regions (UTRs) are provided (only for *S. pombe*), then we use those coordinates. In the four other genomes, the IGR coordinates were treated as the UTR coordinates, with the direction of transcription of the adjacent gene used to designate the UTR as either 5′ or 3′.

Experiments with positive control guanine riboswitch

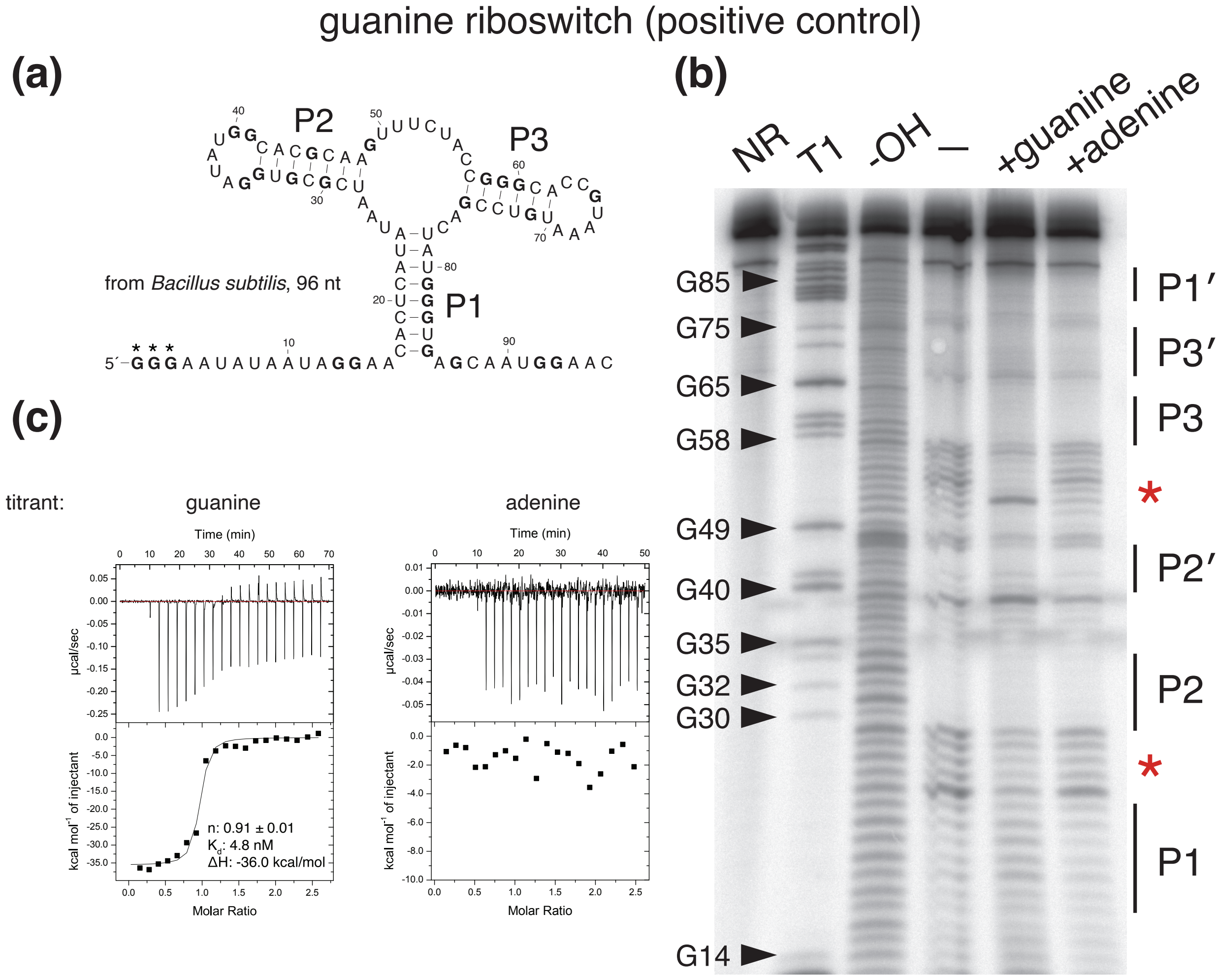

Figure S3: **Experiments with guanine riboswitch**. **(a)** The sequence and structure of the *B. subtilis xpt-pbuX* guanine riboswitch. Guanine residues are bolded. The first three guanine residues (in asterisks) were added to the beginning of the sequence to increase efficiency of in vitro transcription. **(b)** In-line probing of guanine riboswitch: red asterisks indicate the regions of differential degradation between the lane incubated with 10 μM guanine (+guanine) and those incubated with no ligand (-) or 10 μM adenine (+adenine). The no reaction (NR) lane corresponds to undigested, precursor RNA, the T1 lane corresponds to the RNA digested with RNase T1 which cleaves after guanine residues, and -OH lane corresponds to the RNA under partial alkaline digestion, which produces a single nucleotide ladder. Typically, structured regions are less susceptible to degradation, and our gel is concordant with this: the base pairing regions labeled "P1" to "P3" are less frequently the site of degradation than unpaired regions. The regions of differential degradation are also consistent with gels from the literature (2). **(c)** Representative isothermal titration calorimetry results for the guanine riboswitch. The top window in each plot shows the raw ITC data and the bottom window shows ΔH. 1.5 μM RNA was titrated against 15 μM guanine (left) or adenine (right). Only titration with guanine results in the expected sigmoidal binding isotherm and has derived thermodynamic parameters consistent with the literature (3).

Experiments with *GLY1* 3′ UTR and *MET13* 3′ UTR

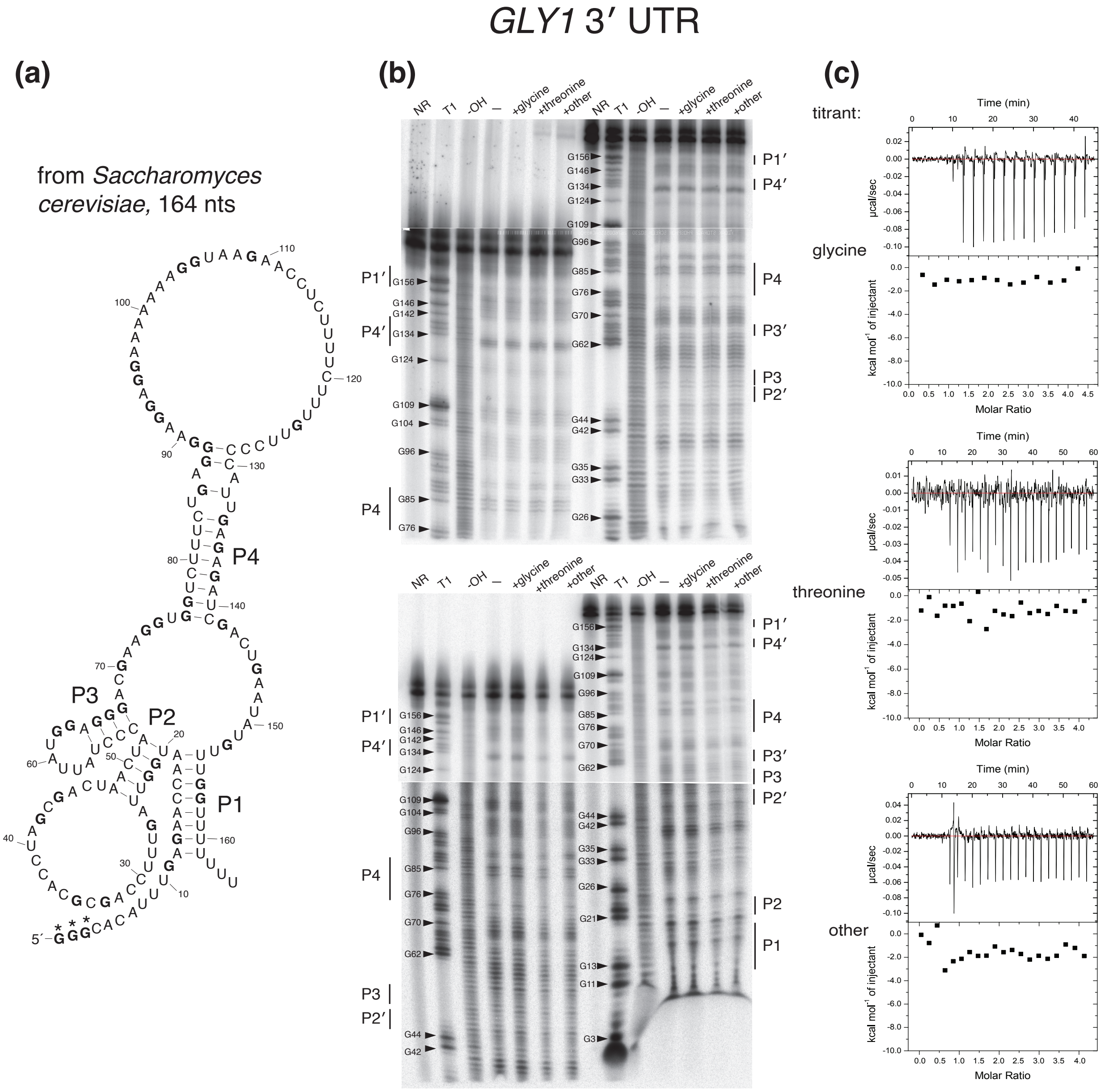

Figure S4: **Experiments with the *GLY1* 3′ UTR structure**. **(a)** The sequence and proposed structure of the *GLY1* 3′ UTR structure from *S. cerevisiae*. Guanine residues are bolded. The first three guanine residues (in asterisks) were added to the beginning of the sequence to increase efficiency of in vitro transcription. **(b)** Two gels from the in-line probing assay are shown. In each case, half of the sample was run initially at 50 W on a sequencing gel. After three hours, the second half was loaded. Therefore, the first half was run for 6 hours total to increase resolution of the 3′ end of the RNA. There is no differential degradation between the RNA incubated with no ligand (-) compared to those incubated with 10 mM glycine, 10 mM L-threonine, or a mixture of several other compounds (L-serine, tetrahydrofolate, L-aspartate, guanine, adenine, PLP, and TPP) all at 10 mM. The no reaction (NR) lane corresponds to undigested, precursor RNA, the T1 lane corresponds to the RNA digested with RNase T1 which cleaves after guanine residues, and the -OH lane corresponds to the RNA under partial alkaline digestion, which produces a single nucleotide ladder. Typically, structured regions are less susceptible to degradation, and our gel is concordant with this: the base pairing regions labeled “P1” to “P4” are less frequently the site of degradation than unpaired regions. **(c)** Representative isothermal titration calorimetry results. The top window in each plot shows the raw ITC data and the bottom window shows ΔH. 5 μM RNA was titrated against 100 μM of either glycine, threonine, or the mixture of other ligands. None resulted in the sigmoidal binding isotherm expected if a ligand were binding to the RNA.

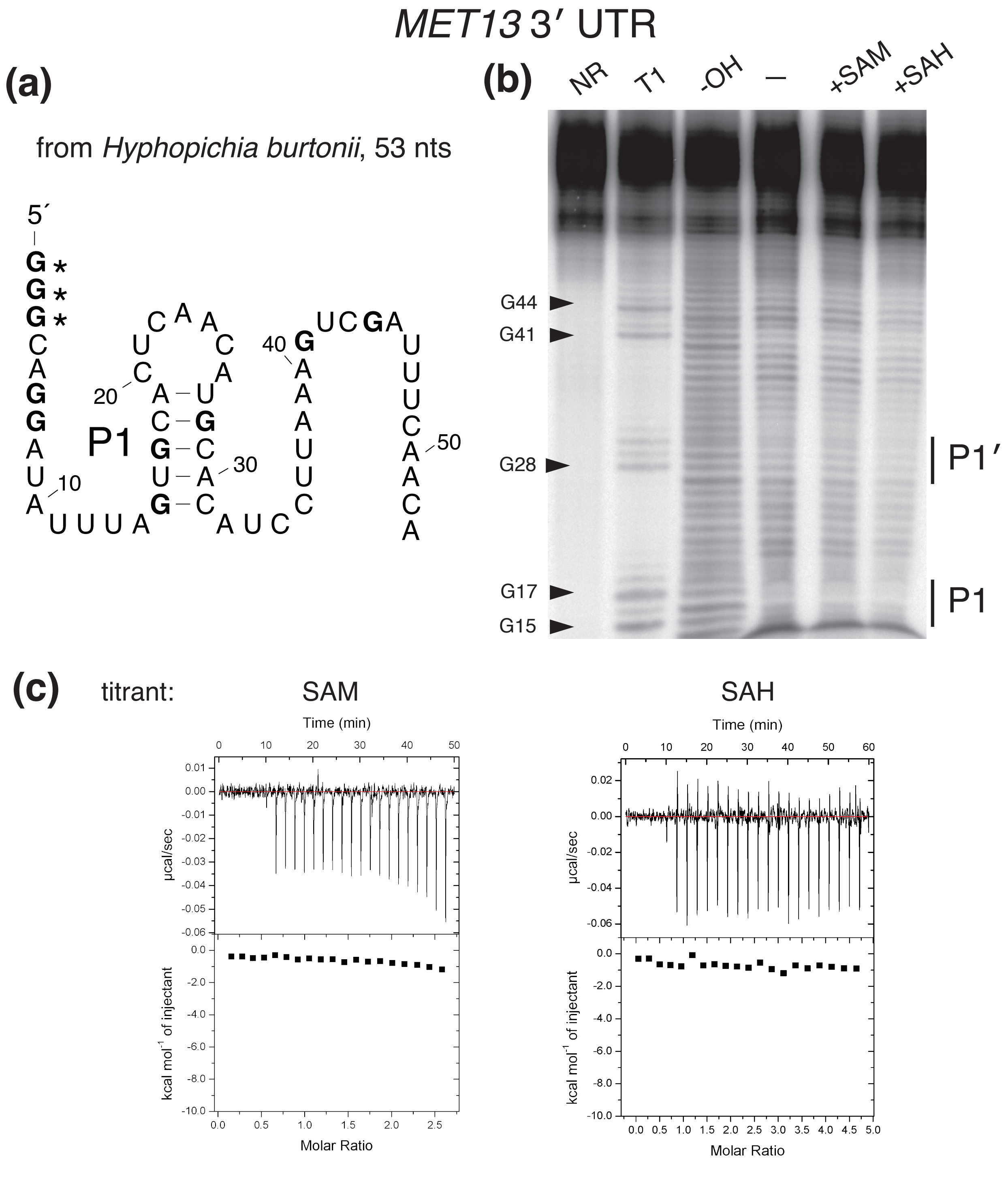

Figure S5: **Experiments with the *MET13* 3′ UTR structure**. **(a)** The sequence and proposed structure of the *MET13* 3′ UTR structure from *H. burtonii*. Guanine residues are bolded. The first three guanine residues (in asterisks) were added to the beginning of the sequence to increase efficiency of in vitro transcription. **(b)** Results from the in-line probing assay are shown. There is no differential degradation between the RNA incubated with no ligand (-) compared to those incubated with 250 μM SAM or SAH. The no reaction (NR) lane corresponds to undigested, precursor RNA, the T1 lane corresponds to the RNA digested with RNase T1 which cleaves after guanine residues, and the -OH lane corresponds to the RNA under partial alkaline digestion, which produces a single nucleotide ladder. **(c)** Representative isothermal titration calorimetry results. The top window in each plot shows the raw ITC data and the bottom window shows ΔH. On the left, 12 μM RNA was titrated against 150 μM SAM and on the right 8 μM RNA was titrated against 180 μM SAH. Neither resulted in the sigmoidal binding isotherm expected if a ligand were binding to the RNA.

Supplemental Information

Fungal genomes include in database

A list in alphabetical order of the 1371 Ascomycota fungal genomes in the whole genome database used for homology search is included.

High-quality genome annotations used to determine if structures are associated with mRNAs

To determine whether a structural ncRNA candidate was located within an UTR or intron of mRNA, we examined if its representatives were consistently located adjacent or within the annotated mRNA of that gene, respectively. The following 24 relatively complete annotations from Genbank were used for this purpose. For all candidate structures, the genomic coordinates of any representatives from a genome in this list were used to determine its genomic context.

Ascodesmis nigricans, *Aspergillus fumigatus*, *Aspergillus oryzae*, *Candida albicans*, *Candida viswanathii*, *Debaryomyces hansenii*, *Eremothecium gossypii*, *Fusarium verticillioides*, *Kazachstania africana*, *Kluyveromyces lactis*, *Lachancea thermotolerans*, *Naumovozyma dairenensis*, *Neosartorya fischeri*, *Neurospora crassa*, *Penicillium chrysogenum*, *Purpureocillium lilacinum*, *Rasamsonia emersonii*, *Saccharomyces cerevisiae*, *Scheffersomyces stipitis*, *Schizosaccharomyces pombe*, *Talaromyces stipitatus*, *Thermothelomyces thermophilus*, *Torrubiella hemipterigena*, and *Uncinocarpus reesii*.

Structures we have classified as within UTRs or introns are consistent with those locations in all of the above genomes that have homologs. For example, structure 11 is found in 7 of these genomes. We designated it as associated with the lysyl-tRNA synthetase because it is found in an intron of that gene in *A. fumigatus*, *A. oryzae*, *P. chrysogenum*, *T. stipitatus*, and *T. hemipterigena*, and in its 5′ UTR in *N. fischeri* and *P. lilacinum*.

Alignments of structural ncRNA candidates

The alignments of the 17 structural ncRNAs are available in a zip folder. The alignments are in Stockholm format, the file format used by all software included in our method (HMMER, Infernal, and R-scape).

In some cases, running R-scape (v1.5.16) with the -s –fold options will produce structures that appear slightly different than those presented in the main figures. In most cases, this is because we individually trimmed the R2R renderings of some structures to include only the regions with conserved structure.

For H/ACA structures 2 and 3, the alignment resulting from our method produced many possibly spurious covariations, likely because the sequences were initially aligned using nhmmer, which generates an alignment based only on primary sequence conservation. This issue is described in more detail in the section "Misalignments can induce spurious covariations" from the preprint "Evolutionary conservation of RNA sequence and structure," available at http://rivaslab.org/

publications.html. For those two cases only, a covariance model was built using the proposed structure after iterative nhmmer homology search and a single sequence from a model organism (*C. albicans* for structure 2 and *A. fumigatus* for structure 3). This covariance model was calibrated and used to search against the hits from the initial alignment, with an E-value of 10^{-5}, to produce a more structurally-informed alignment. R-scape -s –fold was then run on this alignment and displayed in the figures shown here.

Sequences used in experiments

For guanine riboswitch

Before using in-line probing and isothermal titration calorimetry on our experimental RNAs, we first performed these assays on a positive control, the guanine riboswitch.

The following sequence from the *B. subtilis xpt-pbuX* guanine riboswitch was placed in pUC19 plasmid between the BamHI and EcoRI restrictions downstream of the T7 RNA Polymerase promoter sequence (5′-TAATACGACTCACTATAG-3′). Three Gs were added directly after the promoter sequence to enhance efficiency of transcription.

5′-AATATAATAGGAACACTCATATAATCGCGTGGATATGGCACGCAAGTTTCTA CCGGGCACCGTAAATGTCCGACTATGGGTGAGCAATGGAAC-3′

Prior to in vitro transcription, PCR amplification of the plasmid was performed using the following primers: a forward primer (5′-GCTATGACCATGATTACG-3′) that anneals upstream in the pUC19 backbone and a reverse primer (5′-GTTCCATTGCTCACCCATAG-3′) that is the reverse complement of the end of the inserted sequence.

For the *GLY1* 3′ UTR structure

The following sequence corresponding to the *GLY1* 3′ UTR structure from *S. cerevisiae* was placed in pUC19 plasmid between the BamHI and EcoRI restriction sites downstream of the T7 RNA Polymerase promoter sequence. Three Gs were added directly after the promoter sequence to enhance efficiency of transcription.

5′- CACATTTGAGAACCAATGGTTAGTTTCCAGCGCACCTAGCGACTAACTACCCTA TTATGGAGGGACGAAGGTGGTCTTTCTGAGGGAAGGAGGAAAAAAAGGTAAGAACCT CTTTTCTTTGTTCCCCATTGAGAGATCGACTGAATATGTTTGGTTTTTTTGG-3′

Prior to in vitro transcription, PCR amplification of the plasmid was performed using the following primers: a forward primer (5′-GCTATGACCATGATTACG-3′) that anneals upstream in the pUC19 backbone and a reverse primer (5′-GAATTCCCAAAAAAACCAAACA-3′) that is the reverse complement of the EcoRI restriction site (3′-CTTAAG-5′) and end of the inserted sequence.

For the *MET13* 3′ UTR structure

The following sequence corresponding to the *MET13* 3′ UTR structure from *Hyphopichia burtonii* was synthesized as an oligonucleotide. Because the stem has only 5 base pairs, this was chosen

instead of the *C. albicans* sequence which contains a wobble base pair that may be less stable in vitro.

Its sequence is: 5′-CAGGATATTTAGTGCACTCAACATGCACATCCTTAAAGTCGATTTC AACA-3′. PCR amplification was performed using a forward primer (5′-TAATACGACTCACTATA GGGCAGGATATTTAGTGCACTCAAC-3′) which contains the T7 RNA Polymerase promoter, three Gs to increase transcriptional efficiency, and a 22-nucleotide region identical to the start of the desired amplified sequence. The reverse primer used was 5′-TGTTGAAATCGACTTTAAGGATG TG-3′.

References

1. Ares Jr, M. (1986) U2 RNA from yeast is unexpectedly large and contains homology to vertebrate U4, U5, and U6 small nuclear RNAs. *Cell*, **47**, 49–59.

2. Mandal, M., Boese, B., Barrick, J.E., Winkler, W.C., and Breaker, R.R. (2003) Riboswitches control fundamental biochemical pathways in *Bacillus subtilis* and other bacteria. *Cell*, **113**, 577–586.

3. Gilbert, S.D., Stoddard, C.D., Wise, S.J., and Batey, R.T. (2006) Thermodynamic and kinetic characterization of ligand binding to the purine riboswitch aptamer domain. *Journal of Molecular Biology*, **359**, 754–768.
