# Supplementary material for: Discovery of 17 conserved structural RNAs in fungi
Source: Nucleic Acids Res. 2021 Jun 4;49(11):6128–43. doi: 10.1093/nar/gkab355 (PMC8216456; doi:10.1093/nar/gkab355)
Supplement: gkab355_Supplemental_Files [file gkab355_supplemental_files.zip › supplemental_data.tar.gz!/supplemental_data/supplement/figures/figure_S2.pdf]

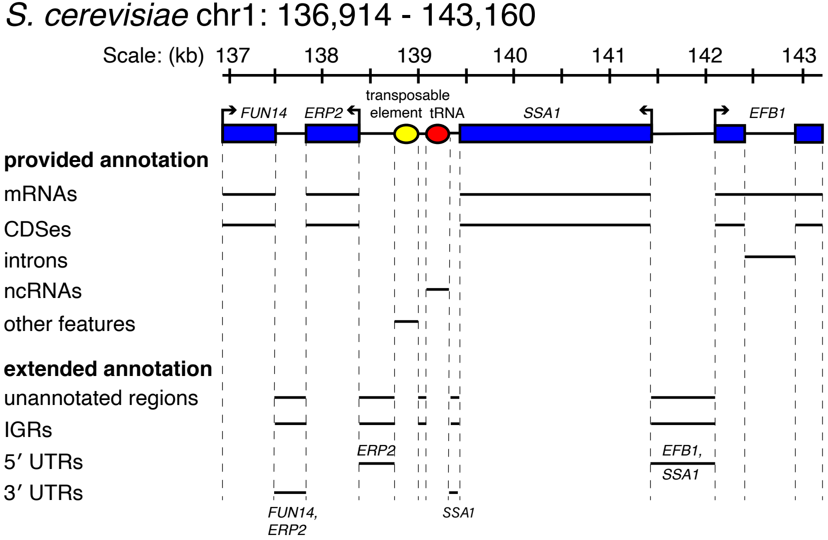

S. cerevisiae chr1: 136,914 - 143,160
Scale: (kb) 137 138 139 140 141 142 143
FUN14
ERP2
transposable element
tRNA
SSA1
EFB1
provided annotation
mRNAs
CDSes
introns
ncRNAs
other features
extended annotation
unannotated regions
IGRs
5′ UTRs
3′ UTRs
ERP2
EFB1, SSA1
FUN14, ERP2
SSA1
