# Supplementary material for: Discovery of 17 conserved structural RNAs in fungi
Source: Nucleic Acids Res. 2021 Jun 4;49(11):6128–43. doi: 10.1093/nar/gkab355 (PMC8216456; doi:10.1093/nar/gkab355)

positive control screen

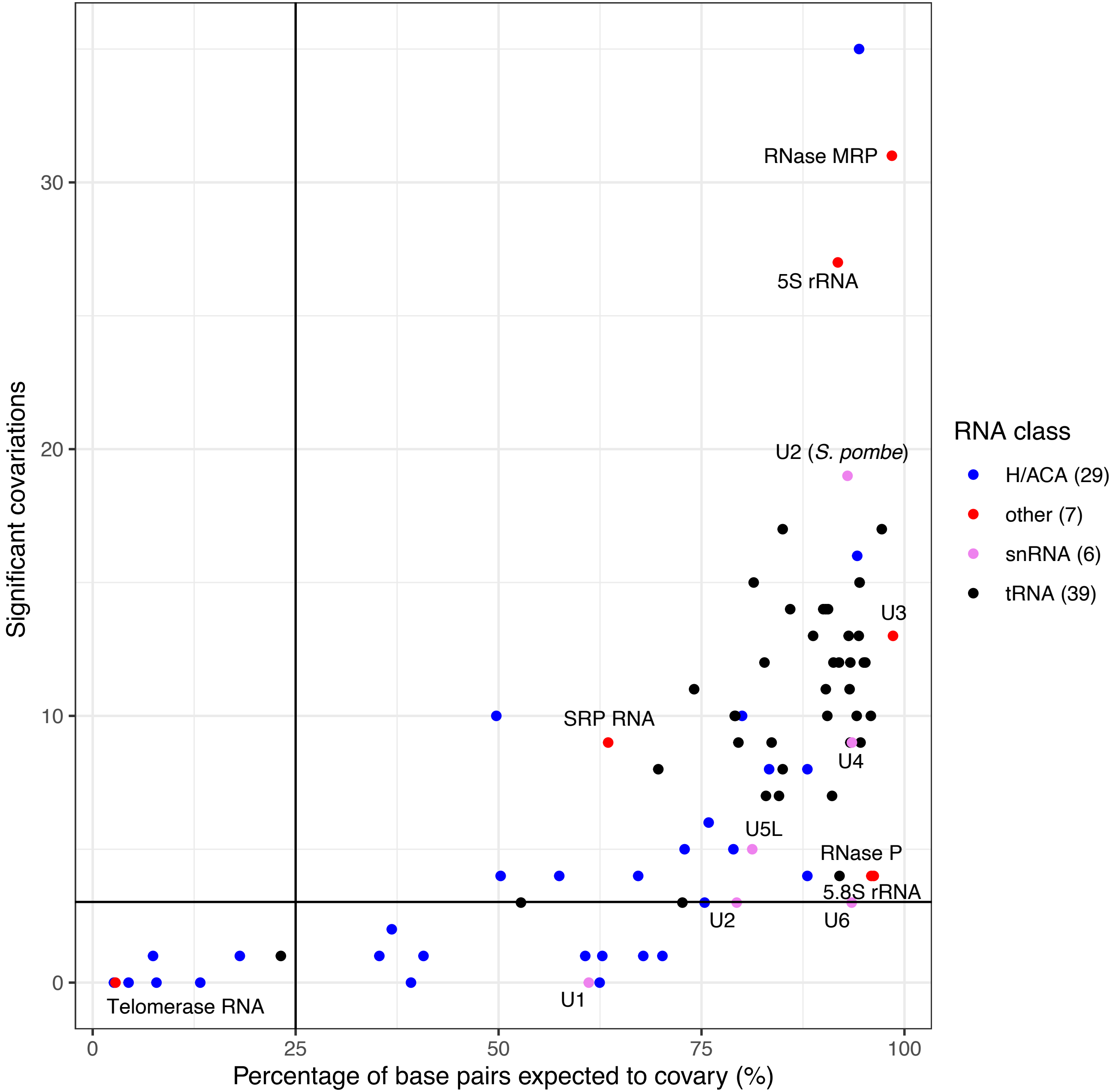

Supplement: gkab355_Supplemental_Files [file gkab355_supplemental_files.zip › supplemental_data.tar.gz!/supplemental_data/supplement/figures/figure_S1.pdf]
