# Supplementary material for: Discovery of 17 conserved structural RNAs in fungi
Source: Nucleic Acids Res. 2021 Jun 4;49(11):6128–43. doi: 10.1093/nar/gkab355 (PMC8216456; doi:10.1093/nar/gkab355)
Supplement: gkab355_Supplemental_Files [file gkab355_supplemental_files.zip › supplemental_data.tar.gz!/supplemental_data/supplement/figures/figure_S5.pdf]

MET13 3′ UTR

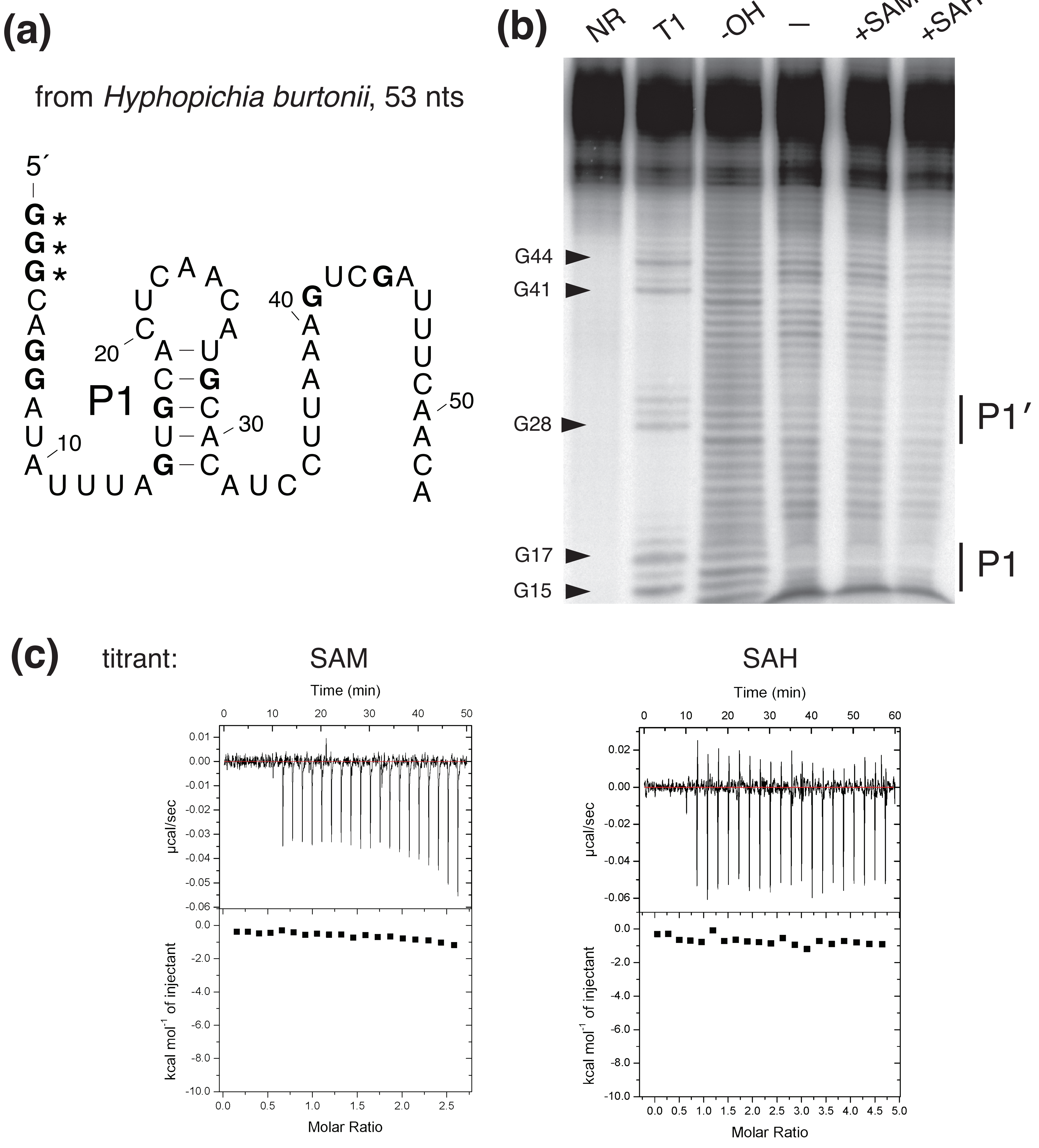
(a)
from Hyphopichia burtonii, 53 nts
5′
P1
10
20
30
40
50
(b)
NR
T1
-OH
—
+SAM
+SAH
G44
G41
G28
G17
G15
P1′
P1
(c)
titrant:
SAM
SAH
Time (min)
μcal/sec
kcal mol-1 of injectant
Molar Ratio
