# Supplementary material for: Discovery of 17 conserved structural RNAs in fungi
Source: Nucleic Acids Res. 2021 Jun 4;49(11):6128–43. doi: 10.1093/nar/gkab355 (PMC8216456; doi:10.1093/nar/gkab355)
Supplement: gkab355_Supplemental_Files [file gkab355_supplemental_files.zip › supplemental_data.tar.gz!/supplemental_data/supplement/figures/figure_S4.pdf]

GLY1 3′ UTR

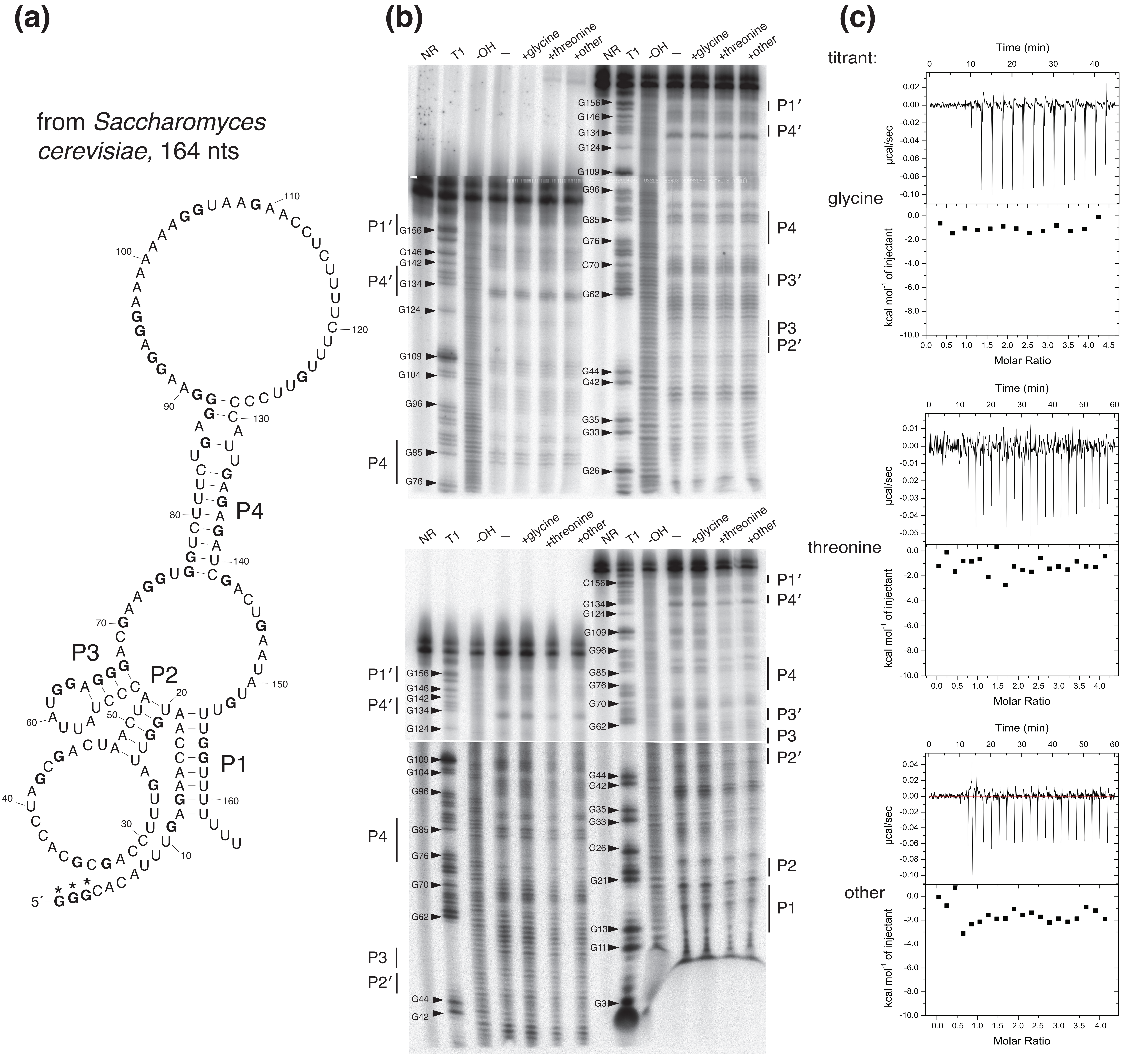
(a)
from *Saccharomyces cerevisiae,* 164 nts
P1
P2
P3
P4
5′
(b)
NR
T1
-OH
–
+glycine
+threonine
+other
P1′
P4′
P4
P3′
P3
P2′
P2
P1
(c)
titrant:
glycine
threonine
other
Time (min)
µcal/sec
kcal mol^{-1} of injectant
Molar Ratio
